# METTL14 Promotes Lipid Synthesis in Dairy Goat Mammary Epithelial Cells by Targeting *CEBPB* via m6A-YTHDF1/3-Dependent Manner

**DOI:** 10.3390/ani16020181

**Published:** 2026-01-08

**Authors:** Hongyun Jiao, Lu Zhu, Xinyu Tang, Ping Gong, Wei Wang, Baolong Liu, Jun Luo

**Affiliations:** 1 Shaanxi Key Laboratory of Molecular Biology for Agriculture, College of Animal Science and Technology, Northwest A&F University, Yangling, Xianyang 712100, China; hongyunj@nwafu.edu.cn (H.J.);; 2 Institute of Animal Husbandry Quality Standards, Xinjiang Academy of Animal Sciences, Urumchi 830011, China

**Keywords:** m6A, METTL14, lipid metabolism, dairy goats

## Abstract

N6-methyladenosine (m6A), a predominant and reversible modification of mammalian RNA, plays a critical role in regulating growth, development, and metabolism. While methyltransferase-like 14 (METTL14) is an essential component of the m6A methyltransferase complex, its specific function in regulating milk fat metabolism in dairy goats remains unexplored. This study therefore aimed to elucidate the role of METTL14 in lipid metabolism within dairy goat mammary epithelial cells (GMECs). METTL14 overexpression significantly promoted the synthesis of TAG (Triacylglycerol) and TC (Total cholesterol), as well as lipid droplet accumulation in GMECs. Furthermore, METTL14 upregulated CCAAT enhancer binding protein beta (CEBPB) expression at both the mRNA and protein levels by directly inducing m6A modification on its transcripts. Finally, we confirmed that m6A modification occurs specifically at site 1662 of *CEBPB* mRNA, and the “Readers” YTH N6-methyladenosine RNA binding protein F1 and F3 (YTHDF1/3) were found responsible for the m6A site recognition and interpretation. This study demonstrated that METTL14 facilitates lipid synthesis and deposition in GMECs. Mechanistically, METTL14 installs the m6A modification at site 1662 of *CEBPB* transcripts. This m6A mark is specifically recognized by the readers YTHDF1 and YTHDF3, which promote the translation of *CEBPB* mRNA, thereby upregulating its expression.

## Simple Summary

As one of the key methyltransferases of m6A, METTL14 exhibited a regulatory role in fat metabolism in previous studies. However, direct functional validation of its role in milk fat metabolism of dairy goats is currently lacking. Our study elucidates how METTL14, a core “Writer” of m6A RNA methylation modification, regulates milk fat synthesis in dairy goats. By adjusting METTL14 level in goat mammary epithelial cells, we demonstrated that METTL14 overexpression boosts triglyceride and cholesterol biosynthesis and lipid droplet accumulation, while reducing METTL14 suppresses these metabolic processes. Mechanistically, METTL14 mediates m6A modification at a specific site of *CEBPB* transcripts, which is subsequently recognized by m6A “Readers” YTHDF1/3, leading to the activation of lipogenic programs. These findings establish an epigenetic paradigm for m6A-mediated regulation of milk fat synthesis and propose novel strategies for optimizing dairy production through molecular breeding.

## 1. Introduction

According to FAO statistics, the global population of dairy goats stood at 219 million, with a total milk production of approximately 20,725,281 tons, accounting for 2.26% of global milk production [1]. Despite its relatively modest market share, goat milk has garnered consumer interest due to its various nutritional and functional advantages. Renowned for its ease of digestion, goat milk has also been reported to possess antibacterial and anti-inflammatory properties [2]. The lipid components of goat milk play a crucial role in these beneficial effects. For instance, smaller fat globule size and higher content of short- and medium-chain fatty acids in goat milk contribute to its enhanced digestibility [3]. Furthermore, goat milk is notably rich in polyunsaturated fatty acids (PUFAs), linked to a reduced risk of cardiovascular diseases [4]. Consequently, this has sparked continuous research and exploration into milk lipid metabolism in dairy goats.

Methylation constitutes approximately 60% of all types of RNA modifications [5], with m6A being the typical form. As a critical epigenetic modification, m6A is the most prevalent, abundant, and conserved internal co-transcriptional modification in eukaryotic RNA, particularly in higher eukaryotic cells [6]. m6A is a reversible methylation modification that occurs at the sixth nitrogen atom of adenylate ribonucleotide. This modification is catalyzed by the methyltransferase complex, known as “Writers,” and can be reversed by the demethylase “Erasers.” The altered methylation status is then recognized by “Readers,” proteins that transmit this signal to downstream processes, thereby influencing the post-transcriptional state of mRNA. The m6A modification mediates various RNA biological processes, including transcription, splicing, maturation, stability, translation, and degradation, thus affecting cell growth, metabolism, and apoptosis [7,8].

The m6A methylation process is regulated by a protease complex that primarily contains two key enzymes: METTL3 and METTL14 [9,10]. METTL14 not only provides structural support for METTL3 but also plays a critical role in m6A mRNA recognition and binding [11]. Functionally, METTL14 is involved in various biological processes, including cancer proliferation and migration, as well as lipid metabolism [12,13]. While for another key component of the m6A complex, METTL3, its role in the regulation of milk protein and fat synthesis in cows has been demonstrated [14], functional data for METTL14 in the mammary gland remain lacking. Consequently, the mechanism by which METTL14 regulates milk fat metabolism in dairy goats remains elusive. Given the complex and multi-level regulation of milk fat synthesis, a comprehensive understanding requires investigation not only at the transcriptional but also at the post-transcriptional level.

In this study, we aim to elucidate the regulatory role of METTL14 in lipid metabolism in goat mammary epithelial cells. We hypothesized that METTL14, as a key m6A methyltransferase, regulates lipid synthesis in GMECs through m6A modification of transcripts of key lipogenesis genes. And we identified the novel target gene of METTL14 that is involved in lipid metabolism and also pinpointed its m6A modification site. Therefore, this finding provides insights into the mechanisms of how METTL14-mediated post-transcriptional modification regulates mammary lipid synthesis in dairy goats.

## 2. Materials and Methods

### 2.1. Cell Culture and Treatment

Primary goat mammary epithelial cells (GMECs) were isolated from the mammary tissue of healthy, three-year-old Xinong Saanen dairy goats at mid-lactation (150 days post-parturition), as previously described by He et al. [15]. GMECs were cultured in basal media at 37 °C, in an atmosphere of 5% CO_2_. The basal media contain the following components: 90% DMEM/F12 (C11330500BT, Gibco, Grand Island, NY, USA), 10% fetal bovine serum (A5256701, Gibco, Grand Island, NY, USA), 5 μg/mL hydrocortisone (H0888, Sigma-Aldrich, St. Louis, MO, USA), 100 U/mL penicillin/streptomycin (Pharmaceutical Group, Harbin, China), 10 ng/mL epidermal growth factor (PHG0311, Invitrogen, Carlsbad, CA, USA). When cell density reached 75%, lipofectamine 3000 (L3000015, Invitrogen, Carlsbad, CA, USA) was used to transfect reconstructed vectors, while RNAiMAX (13778150, Invitrogen, Carlsbad, CA, USA) was used to transfect siRNA (GenePharma, Shanghai, China) into cells, per manufacturer’s instructions. Cells were collected after 24 h of transfection for qPCR and lipid analysis, 48 h post-transfection for Western blot analysis.

### 2.2. Overexpression Vector Construction

The coding sequence of *METTL14* gene of dairy goat was downloaded from NCBI (XM_005681276.3). Primers were designed by Primer Premier 5, sense: 5′-CCCAAGCTTATGGACAGCCGCTTGCAGGA-3′; anti-sense: 5′-GGGGTACCCTATCGAGGTGGAAAGCCACC-3′. The coding sequence of *METTL14* was inserted into pcDNA3.1 between the *HindIII* and *KpnI* restriction sites.

### 2.3. mRNA Analysis

mRNA extraction and analysis were carried out as previously described by Huang et al. [16]. The primers used in this study are included in Table S1. The relative gene transcriptional levels were calculated as 2^−∆∆CT^, normalized to the housekeeping gene *UXT*.

### 2.4. m6A Content Quantification

Total RNA was extracted as previously described by Huang et al. [16]. The quantity of m6A RNA was determined by EpiQuik m6A RNA Methylation Quantification Kit (Colorimetric) (P-9005, Epigentek, Farmingdale, NY, USA) following the manufacturer’s instruction. 200 ng RNA was used for each test. The amount of m6A RNA was eventually quantified by a microplate spectrophotometer at a wavelength of 450 nm (BioTek, San Diego, CA, USA).

### 2.5. m6A RNA Immunoprecipitation Followed by Quantitative Polymerase Chain Reaction

Total RNAs were then sonicated and the fragments containing m6A modification were collected using the EpiQuik CUT&RUN m6A RNA Enrichment (MeRIP) Kit (P-9018, Epigentek, Farmingdale, NY, USA) via immunoprecipitation. Briefly, 10 μg total RNA was cleaved and then incubated with magnetic beads at room temperature for 90 min. Beads were then collected and washed three times with 90% ethanol. The beads were resuspended in 13 µL of elution buffer and incubated at room temperature for 5 min to release RNAs. The purified RNAs were then reverse-transcribed and quantified via qPCR as described above.

### 2.6. Western Blot Analysis

Western blot analysis was conducted as described by Zhu et al. [17]. The primary antibodies include METTL14 (26158-1-AP, Proteintech, Rosemont, IL, USA, 1:1000), CEBPB (23431-1-AP, Proteintech, Rosemont, IL, USA, 1:1000), and β-actin (CW0096, CW Biotech, Beijing, China, 1:1000). The secondary antibodies include Horseradish peroxidase (HRP)-conjugated goat antirabbit IgG (CW0103, CW Biotech, Beijing, China, 1:4000) and polyclonal goat anti-mouse HRP-conjugated IgG (CW0102, CW Biotech, Beijing, China, 1:4000).

### 2.7. Cellular Triacylglycerol and Total Cholesterol Assay

Triglyceride (TAG) enzymatic assay kit (E1013, Applygen, Beijing, China) and total cholesterol (TC) enzymatic assay kit (E1005, Applygen, Beijing, China) were used to measure the content of cellular triacylglycerol and cholesterol. Cells were washed with cold phosphate-buffered saline (PBS) and lysed with lysis buffer. After centrifugation, a portion of the supernatant was heated at 70 °C for 10 min, which was then used for the enzymatic assays. Another portion was used for protein quantification by the bicinchoninic acid (BCA) protein assay kit (A55860, Thermo Fisher, Waltham, MA, USA). Eventually, the triglyceride and cholesterol contents were calibrated with the cellular protein levels.

### 2.8. Lipid Droplets Staining

GMECs were washed three times in PBS and fixed with 4% paraformaldehyde (BL539A, Biosharp, Hefei, China) for 1 h. Cells were incubated with 300 μL boron-dipyrromethene (BODIPY) solution (IP4160, Solarbio, Beijing, China) for 30 min to stain cellular lipid droplets and then incubated with 4′,6-diamidino-2-phenylindole (DAPI) (C0065, Solarbio, Beijing, China) for 10 min to stain nuclei. Finally, the relative fluorescence intensity of BODIPY (GFP channel) and DAPI was determined using a fluorescence microscope (Bio Tek, San Diego, CA, USA).

### 2.9. Dual-Luciferase Reporter Assay

Overlap extension polymerase chain reaction (PCR) was used to generate partially overlapping fragments containing the site-directed mutation. The primers used in this study are included in Table S2. The mutant sequence of *CEBPB* was cloned into psi-CHECK2 plasmid between *KpnI* and *XhoI* restriction sites to produce the reconstructed vectors. After 24 h co-transfection of the psi-CHECK2 reconstructed vectors and *METTL14*-pcDNA3.1 plasmid, GMECs were lysed and incubated with luciferase assay buffer. Firefly luciferase activities were quantified by microplate luminometer (5200330, Thermo Fisher, Waltham, MA, USA) as F-values. Then, Stop & Glo Reagent was added immediately, and renilla luciferase activities were determined as the R-values. The activity of each mutant was calculated via R-values/F-values.

### 2.10. Statistical Analysis

The independent samples two-tailed Student’s *t*-test was used to analyze the differences between two groups of data. For qPCR data, the expression level of each target gene was treated as an independent comparison between the experimental and control groups. Each experiment was repeated at least three times. The results were considered significant when *p* < 0.05 (* indicates *p* < 0.05; ** indicates *p* < 0.01). Data were exhibited as mean ± standard deviation (SD).

## 3. Results

### 3.1. METTL14 Facilitates the Accumulation of Lipid Droplets in GMECs

**Figure 1:**
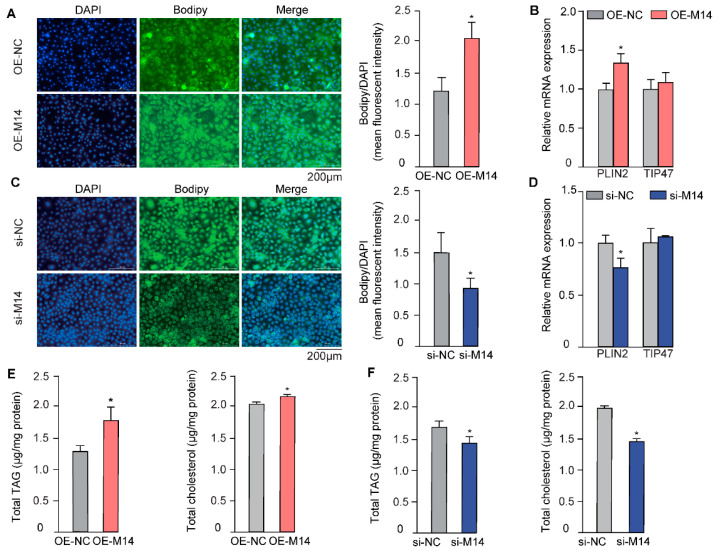
METTL14 promoted lipid synthesis in GMECs. BODIPY staining (**A**) and the relative mRNA expression of lipid droplets accumulation-related gene *PLIN2* and *TIP47* (**B**) upon METTL14 overexpression in GMECs. BODIPY staining (**C**) and the relative mRNA expression of lipid droplets accumulation-related gene *PLIN2* and *TIP47* (**D**) upon METTL14 knockdown in GMECs. Relative cellular total triglyceride and cholesterol levels in GMECs upon METTL14 overexpression (**E**) or knockdown (**F**). (* *p* < 0.05).

Lipid droplets within the mammary epithelium are the major source of milk fat, which are composed of a neutral lipid core enveloped by a phospholipid monolayer, serving as the primary storage form of lipids [18]. Therefore, we first explored the role of METTL14 in lipid droplet formation in GMECs. We successfully manipulated its expression via vector-mediated overexpression or siRNA-induced gene knockdown (Figure S1). Upon overexpression of METTL14, a marked increase in lipid droplet content was observed in GMECs, as revealed by BODIPY staining (Figure 1A). Conversely, the number of lipid droplets was significantly reduced (Figure 1C) when METTL14 was knocked down. Perilipin 2 (PLIN2) and tail-interacting protein (TIP47) are crucial structural components of lipid droplets, which are essential for the formation and maintenance of lipid droplets [19]. Our findings revealed that manipulation of METTL14 expression led to corresponding changes in *PLIN2* expression, while *TIP47* expression showed no alteration (Figure 1B,D).

The core of lipid droplets is primarily composed of triacylglycerols and cholesteryl esters. Consistently, both TAG and TC levels were significantly elevated in cells overexpressing METTL14 (Figure 1E), but decreased upon METTL14 knockdown (Figure 1F). These observations collectively suggest that METTL14 positively regulates lipid accumulation in GMECs.

### 3.2. METTL14 Affects the Expression of Lipid Metabolism-Related Genes

**Figure 2:**
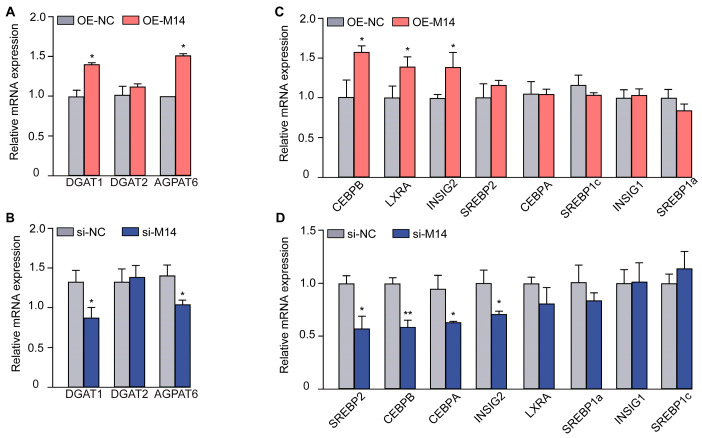
METTL14 affected the expression of genes related to fat synthesis in GMECs. (**A**). The relative mRNA levels of TAG synthesis-related genes *DGAT1*, *DGAT2*, and *AGPAT6* after METTL14 overexpression. (**B**). The relative mRNA level of the TAG synthesis genes after METTL14 knockdown. (**C**). The relative mRNA level of fat metabolism-related transcriptional factors *CEBPA*, *CEBPB*, *INSIG1*, *INSIG2*, *LXRA*, *SREBP1a*, *SREBP1c*, and *SREBP2* after METTL14 overexpression. (**D**). The relative mRNA level of the fat metabolism-related transcriptional factors after METTL14 knockdown. Statistical significance for each gene was determined by *t*-test between the treatment and its control. (* *p* < 0.05; ** *p* < 0.01).

Diacylglycerol O-acyltransferase 1 (DGAT1), diacylglycerol O-acyltransferase 2 (DGAT2), and 1-Acylglycerol-3-Phosphate O-Acyltransferase 6 (AGPAT6) are key players in TAG synthesis. Notably, overexpression of METTL14 led to a significant upregulation of *DGAT1* and *AGPAT6* levels, as illustrated in Figure 2A. Conversely, in cells with METTL14 knockdown, a marked suppression of *DGAT1* and *AGPAT6* was observed (Figure 2B). To elucidate the mechanism by which METTL14 enhances lipid synthesis in GMECs, we analyzed the expression of transcription factors associated with lipid metabolism. Our findings revealed that overexpression of METTL14 resulted in elevated levels of *CEBPB*, liver X receptor α (*LXRA*), and insulin-induced gene 2 (*INSIG2*), while other lipid synthesis-related transcription factors remained unaffected (Figure 2C). Additionally, the expression of sterol regulatory element binding transcription factor 2 (*SREBP2*), *CEBPB*, CCAAT enhancer binding protein alpha (*CEBPA*), and *INSIG2* was notably downregulated in METTL14 knockdown cells (Figure 2D). These results collectively suggest that METTL14 plays a pivotal role in lipid metabolism in GMECs by modulating the expression of key transcriptional factors.

### 3.3. METTL14 Increases the m6A Content of CEBPB

**Figure 3:**
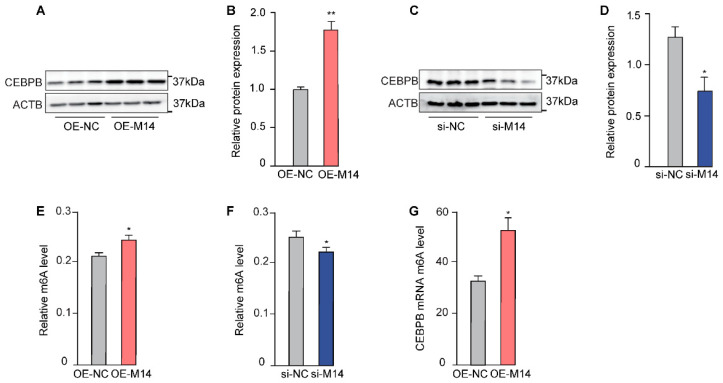
METTL14 induced m6A modification in *CEBPB* transcripts in GMECs. (**A**). CEBPB protein levels upon METTL14 overexpression, which is quantified in (**B**). (**C**). CEBPB protein levels after METTL14 knockdown, which is quantified in (**D**). Cellular total m6A levels were upregulated in METTL14-overexpressed (**E**) but decreased in METTL14-knockdown cells (**F**). (**G**). *CEBPB* mRNA m6A enrichment level (% of input) upon METTL14 overexpression. (* *p* < 0.05; ** *p* < 0.01).

Among all the transcription factors detected, *CEBPB* is the most strongly regulated by METLL14 (Figure 2C,D). Therefore, we selected CEBPB for further investigation. Firstly, we checked the protein level changes of CEBPB upon METTL14 manipulation. Consistent with mRNA levels, the protein level of CEBPB is also positively correlated with the METLL14 expression (Figure 3A–D).

As a crucial component of m6A methyltransferase, METTL14 plays an important role in inducing RNA m6A modification [13]. Not surprisingly, cellular total m6A levels were upregulated in METTL14-overexpressed GMECs (Figure 3E) but decreased after METTL14 knockdown (Figure 3F). We then wondered whether METTL14 could cause m6A modification in *CEBPB* mRNA. As the MeRIP-qPCR result shows, m6A modification of *CEBPB* mRNA was dramatically increased upon overexpression of METTL14 (Figure 3G), indicating *CEBPB* mRNA is a direct target of METTL14.

### 3.4. Identification of the Key m6A Modification Site on CEBPB

**Figure 4:**
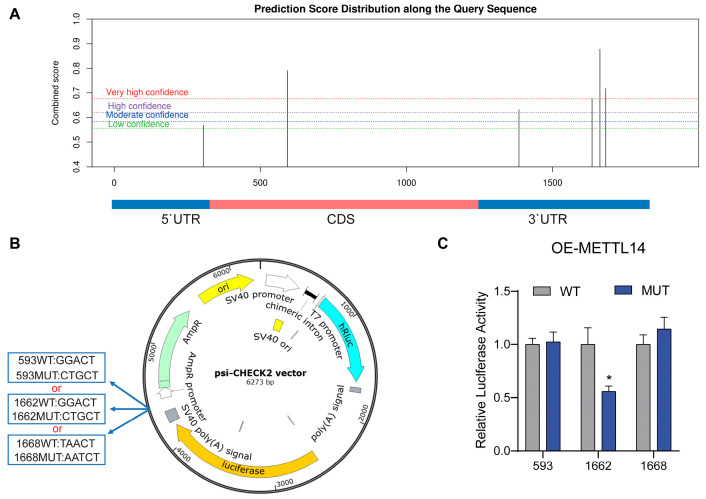
METTL14 regulates *CEBPB* expression through the m6A-modified site. (**A**). The potential m6A modification sites in *CEBPB* mRNA were predicted by SRAMP, and the combined score (*Y*-axis) represents the confidence level of the prediction for each site. (**B**). The diagram of reconstructed psi-CHECK2 plasmids with or without point mutation at sites 593, 1662, and 1668. (**C**). Luciferase activities of GEMCs transfected with each reconstructed psi-CHECK2 plasmid along with METTL14 overexpression. (* *p* < 0.05).

METTL14 only induces m6A modification in the specific sequence of mRNA. An online tool SRAMP [https://www.cuilab.cn/sramp] (accessed on 27 October 2023) was then utilized to predict potential m6A modification sites on the *CEBPB* transcripts [20]. Three predicted sites that exhibited very high confidence scores (Figure 4A) are located at the 593 bp, 1662 bp, and 1668 bp of the 5′ UTR start site of goat *CEBPB* mRNA were thus selected for further validation. Three different mutant sequences, each containing one specific mutant site, were constructed and inserted into the psi-CHECK2 vector, as shown in Figure 4B. Dual luciferase assay was then carried out to identify the modification site. Upon plasmid transfection and METTL14 overexpression in GMECs, only the mutation at site 1662 significantly reduced the luciferase activity (Figure 4C). The results validated site 1662 as the METTL14-induced m6A modification site on *CEBPB* mRNA.

### 3.5. METTL14 Regulates CEBPB in an m6A-YTHDF1/3-Dependent Manner

**Figure 5:**
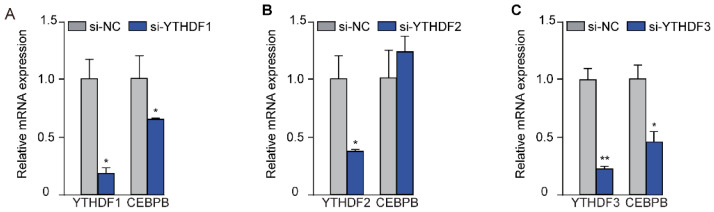
The relative expression of *CEBPB* after interfering the “Readers” of m6A modification. *CEBPB* expression after the transfection of si-*YTHDF1* (**A**), si-*YTHDF2* (**B**), or si-*YTHDF3* (**C**) in GMECs. Statistical significance for each gene was determined by *t*-test between the treatment and its control. (* *p* < 0.05; ** *p* < 0.01).

m6A methylation regulates mRNA transcription through the induction of m6A modification. Meanwhile, “Readers” are required to recognize these m6A marks [21]. YTHDF1, YTHDF2, and YTHDF3 of the YTH N6-Methyladenosine RNA Binding Protein family are well established “Readers.” They recognize and interpret the m6A marks on transcripts, thereby guiding downstream processes such as splicing and translation [22]. To find the “Reader” that is involved in the recognition and interpretation of METTL14-induced m6A marks on *CEBPB* transcripts, we employed siRNA to knock down *YTHDF1*, *YTHDF2*, and *YTHDF3*, followed by the quantification of *CEBPB* mRNA levels. As expected, the designed siRNAs successfully suppressed the expression of *YTHDF1* (Figure 5A), *YTHDF2* (Figure 5B), and *YTHDF3* (Figure 5C). Nevertheless, the expression of *CEBPB* was only markedly decreased in cells treated with si*YTHDF1* and si*YTHDF3*. This observation suggests that “readers” *YTHDF1* and *YTHDF3* are responsible for the recognition of the m6A marks on *CEBPB* transcript, thereby facilitating its translation.

## 4. Discussion

In the present study, we identified that METTL14 promotes lipid synthesis in GMECs through inducing m6A methylation of *CEBPB* transcript at site 1662. Notably, the transcription of *CEBPB*, a pivotal transcription factor associated with lipid synthesis, was found to be regulated by METTL14. Moreover, site 1662 on *CEBPB* transcript was identified as the m6A modification site, which can be further recognized by the “Readers” YTHDF1 and YTHDF3.

As the most prevalent form of RNA modification, m6A methylation is crucial for various biological processes, including the growth and development of mammary tissues. Previous studies have demonstrated that METTL3, a “Writer” of m6A, enhanced milk protein and fat biosynthesis in mammary epithelial cells of dairy cows by inducing m6A modification of *SREBP1* and mTOR signaling pathways [6]. METTL14, another key component of m6A methylation, has also been implicated in the regulation of lipid metabolism in various tissues of different species [23]. For instance, in human hepatocellular carcinoma cells, the knockdown of METTL3/METTL14 resulted in reduced cellular lipid accumulation [24]. In mouse hepatocellular carcinoma cells, METTL14 promoted the translation of *ACLY* and *SCD1* to facilitate fatty acid synthesis and lipogenesis [25]. Under high-fat diet conditions, mice exhibited elevated levels of aortic staining along with increased cholesterol and triglycerides, but these effects were reversed in METTL14 knockdown mice [26]. Additionally, adipocyte-specific METTL14 knockout mice showed significantly lower liver triglyceride content and smaller lipid droplets compared to the control group [27]. These findings collectively suggest that METTL14 promotes lipid synthesis and accumulation in adipose and liver tissues. However, the role of METTL14 in regulating fat synthesis in goat mammary tissues was unexplored.

Given that GMECs are the primary cells responsible for synthesizing and secreting goat milk components, they have been widely used as a cell model to study milk fat synthesis [28]. Consequently, we investigated the role of METTL14 in GMECs. Through genetic manipulation, we found that METTL14 expression positively correlates with lipid droplet formation, TAG, and TC synthesis in GMECs. This finding aligns with previous studies indicating that METTL14 plays a significant role in lipid synthesis. However, considering the complexity of the lipid synthesis network, only a few downstream target genes of METTL14 were identified, such as *ACLY* and *SCD1* [25]. Whether any upstream lipid metabolism-associated transcriptional factors were affected by METTL14 remained unknown.

CEBPB, an essential transcriptional factor regulating lipid metabolism, has been shown to play a key role in lipolysis and lipid synthesis by modulating autophagy, trans-activation of the peroxisome proliferator-activated receptor-γ (PPARγ), and mitotic clonal expansion and differentiation of preadipocytes both in vitro and in vivo [29,30]. The downregulation of CEBPB level significantly inhibits fat synthesis in non-alcoholic fatty liver disease mice [31,32]. In addition, CEBPB knockout mice exhibited a significant reduction in both body weight and hepatic lipid accumulation [33]. CEBPB also plays a crucial role in milk fat metabolism, as studies have shown that it regulates the expression of *FASN*, thereby enhancing milk fat synthesis in mammary epithelial cells of dairy cows [34]. Given the role of CEBPB as a master regulator of lipogenesis, precise control of its expression is critical for lipid homeostasis.

However, the involvement of m6A modification in the transcriptional regulation of CEBPB has not been reported. In our study, RT-qPCR results indicated that the expression of *CEBPB* was significantly upregulated by METTL14 in GMECs. Additionally, MeRIP-qPCR revealed that the m6A level of *CEBPB* mRNA was markedly increased following overexpression of METTL14. METTL14 interacts with METTL3 to form a stable heterodimer in a 1:1 ratio [35]. This heterodimer serves as the catalytic center for adenine methylation by binding to the m6A consensus sequence DRACH (D = A, G, or U; R = G or A; H = A, C, or U) on mRNAs in a co-transcriptional manner [36,37]. Through prediction, we found three highly reliable m6A modification sites in both the coding sequence region and the 3′ UTR of *CEBPB*. Through mutation screening of these sites, we eventually identified that m6A modification occurs at site 1662 in the 3′ UTR region. Critically, our findings highlight METTL14 as a pivotal upstream regulator, robustly upregulating CEBPB via a specific m6A modification.

The YT521-B homology domain family, including YTHDF1, YTHDF2, and YTHDF3, has been demonstrated to be the essential “Readers” for m6A-modified mRNA [36]. Specifically, YTHDF1 binds the m6A site around the stop codon and then enhances the translation of its targets by interacting with initiation factors and facilitating ribosome loading [38]. YTHDF2 promotes mRNA degradation by influencing the stability of m6A-modified mRNA [22]. YTHDF3 affects the translation and decay of methylated mRNAs through cooperation with YTHDF1 and YTHDF2 [38]. Furthermore, recent perspectives have further emphasized the significance and complexity of YTHDF3’s functions. Studies suggest that YTHDF1/2/3 may jointly regulate m6A-mRNA metabolism in a redundant manner [39]. This underscores that YTHDF3 is an indispensable and crucial component within the m6A regulatory network, which supports our experimental results. In this study, the knockdown of *YTHDF1/3* resulted in a significant decrease in *CEBPB* mRNA levels, indicating that they act as “Readers” to regulate the transcriptional level of *CEBPB*. Therefore, our study unveils a novel regulatory mechanism of *CEBPB* mediated by METTL14-induced m6A methylation, providing novel insights into the epigenetic regulation of milk fat synthesis in GMECs.

## 5. Conclusions

This study provides proof-of-principle evidence for the detailed molecular mechanisms underlying METTL14-mediated m6A methylation in regulating goat milk fat synthesis. Specifically, m6A methyltransferase METTL14 regulates the expression of CEBPB through methylation at site 1662 on *CEBPB* mRNA, which is then recognized by the YTHDF1/3 complex, thereby promoting fat deposition in GMECs.

Further in vivo validation is warranted to establish the functional significance of METTL14 in mammary gland development and lactogenesis in dairy goats. Given its pivotal role as an epigenetic regulator, it would be compelling to explore whether environmental modulators-including dietary components, ambient temperature fluctuations, and circadian rhythm-impact METTL14-mediated m6A methylation dynamics in the mammary gland, thereby influencing milk synthesis and composition in dairy goats.

## Data Availability

The original contributions presented in this study are included in the article and Supplementary Materials. Further inquiries can be directed at the corresponding authors.

## Supplementary Materials

The following supporting information can be downloaded at: https://www.mdpi.com/article/10.3390/ani16020181/s1, Table S1: the primers used in this study; Table S2: Primers of CEBPB m6A modification sites mutation; Figure S1: overexpression or knockdown of METTL14 in GMECs.

## Abbreviations

The following abbreviations are used in this manuscript:

m6A	N6-methyladenosine
METTL14	Methyltransferase-like 14
GMECs	Goat mammary epithelial cells
CEBPB	CCAAT enhancer binding protein beta
TAG	Triacylglycerol
TC	Total cholesterol
PBS	Phosphate-buffered saline
BCA	Bicinchoninic acid
BODIPY	Boron-dipyrromethene
DAPI	4′,6-diamidino-2-phenylindole
PCR	Polymerase chain reaction
PUFAs	Polyunsaturated fatty acids
MeRIP-qPCR	RNA methylation immunoprecipitation qPCR
PLIN2	Perilipin 2
TIP47	Tail-interacting protein
DGAT1	Diacylglycerol O-acyltransferase 1
DGAT2	Diacylglycerol O-acyltransferase 2
AGPAT6	Glycerol-3-phosphate acyltransferase 4
LXRA	Liver X receptor α
INSIG2	Insulin induced gene 2
SREBP2	Sterol regulatory element binding transcription factor 2
CEBPA	CCAAT enhancer binding protein alpha
YTHDF1/2/3	YTH N6-methyladenosine RNA binding protein F1/2/3
