# Supplementary material for: METTL14 Promotes Lipid Synthesis in Dairy Goat Mammary Epithelial Cells by Targeting CEBPB via m6A-YTHDF1/3-Dependent Manner
Source: Animals (Basel). 2026 Jan 8;16(2):181. doi: 10.3390/ani16020181 (PMC12838069; doi:10.3390/ani16020181)
Supplement: Supplementary file 1 [file animals-16-00181-s001.zip › animals-4060354-supplementary.pdf]

Supplementary Files

Table S1: Characteristics of Primers Used in the RT-qPCR Reaction

Gene	Accession	Primer Sequences (5’-3’)	Size(bp)	Annealing Temperature (°C)
UXT	NM_001037471	F: TGTGGCCCTTGGATATGGTT<br>R: GGTTGTCGCTGAGCTCTGTG	125	55
CEBPA	XM_018062278.1	F: CTCCGGATCTCAAGACTGCC<br>R: CCCCTCATCTTAGACGCACC	125	55
CEBPB	XM_018058020.1	F: GCCTGTCCACGTCCTCGTCGTCCAGC<br>R: CGGATCTTGTACTCGTCGCTGTGCTTGTCC	169	55
DGAT1	XM_005688895.1	F: CCACTGGGACCTGAGGTGTC<br>R: GCATCACCACACACCAATTCA	101	55
DGAT2	BT030532.1	F: CATGTACACATTCTGCACCGATT<br>R: TGACCTCCTGCCACCTTTCT	100	55
INSIG1	XM_589325	F: AGCCTCACAAGTTCAAGCG<br>R: ACAGTGCTGCTAATGTCAAGG	132	55
INSIG2	XM_614207	F: TCCAGTGTGATGCGGTGTGTA<br>R: TGGATAGTGCAGCCAGTGTGA	109	55
LXRA	GU332719	F: CATCAACCCCATCTTCGAGTT<br>R: CAGGGCCTCCACATATGTGT	163	55
METTL14	XM_0056	F: AGAGAAATTGGCGCAAGGGT	82	55

	81276.3	R: ACTTTCAGCTCCCAACTGCT		
SREBP1a	HM443643.1	F: CTGCTGACCGACATAGAAGACAT<br>R: GTAGGGCGGGTCAAACAGG	81	55
SREBP1c	HM443643.1	F: ACGCCATCGAGAAACGCTAC<br>R: GTGCGCAGACTCAGGTTCTC	181	55
SREBP2	NC_030812.1	F: CGGCGTGATCGTGCTGAGCGTC<br>R: CCGACAGGCAGGTTTGCAGGTTGG	173	55
YTHDF1	XM_018057562.1	F: CGGAGACCACCACTTCGTACAC<br>R: CTTTGAGCCTGCTGACCCTGAG	150	55
YTHDF2	XM_018057048.1	F: CCACTACCAACAGCAGAGCCTAC<br>R: TAAAGGCATAGAGACGGGCG	81	55
YTHDF3	XM_018058464.1	F: GGCTGGATTTGGCAATGATACTCTG<br>R: CCACTGCTGCTCAATGCTGTTC	138	55

Table S2: Primers of CEBPB m6A modification sites mutation

Name	Primer Sequences (5'-3')	Annealing Temperature (°C)
CEBPB-593 MUT	F: AACCTCGAGTACTACGAGGCGGACTGCTT	65
	R: AAAGCGGCCGCCTCCTCCTTCCGCTTGCA	65
CEBPB-1662 MUT	F: AAACTCGAGCAGAACTTTGGCACTGGGGC	60
	R: AGCGCGGCCGCAAAGGCTTTTTAAACATTCT	60
CEBPB-1668 MUT	F: AAACTCGAGCAGAACTTTGGCACTGGGGC	60
	R: AGCGCGGCCGCAAAGGCTTTTTAAACATTCT	60

Figure S1: Overexpression and knockdown of METTL14 in goat mammary epithelial cells

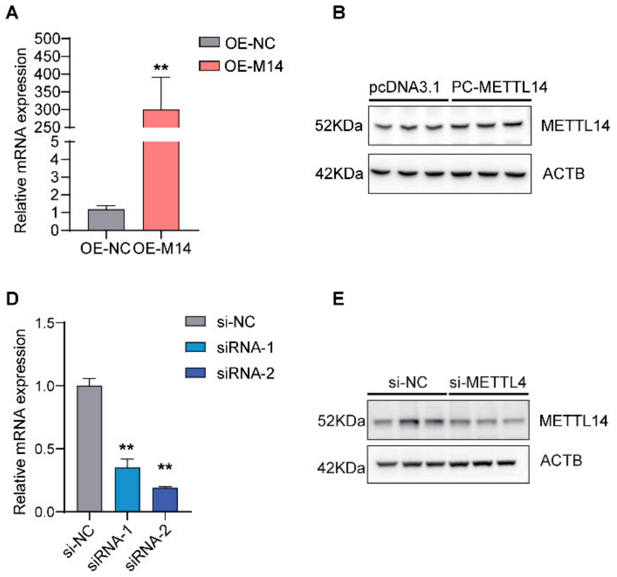

Figure S1: Overexpression and knockdown of METTL14 in goat mammary epithelial cells. The relative mRNA level (A) and protein level (B, C) of METTL14 after transfecting METTL14-pcDNA3.1 plasmid in GMECs. The relative mRNA level (D) and protein level (E, F) of METTL14 after transfecting METTL14 siRNA in GMECs. (* $p < 0.05$; ** $p < 0.01$). OE: Overexpression
